# Stimulating angiogenesis mitigates the unloading-induced reduction in osteogenesis in early-stage bone repair in rats

**DOI:** 10.14814/phy2.12335

**Published:** 2015-03-16

**Authors:** Takeshi Matsumoto, Shota Sato

**Affiliations:** Bioengineering Division, Osaka University Graduate School of Engineering ScienceToyonaka, Japan

**Keywords:** Angiogenic vasculature, bone regeneration, drill-hole defect, mechanical unloading

## Abstract

Accelerating fracture healing during bed rest allows early mobilization and avoids prolonged fracture healing times. We tested the hypothesis that stimulating angiogenesis with deferoxamine (DFO) mitigates the unloading-induced reduction in early-stage bone repair. Rats aged 12 weeks were subjected to cortical drilling on their tibial diaphysis under anesthesia and treated with hindlimb unloading (HU), HU and DFO administration (DFOHU), or weight bearing (WB) for 5 or 10 days (HU5/10, DFOHU5/10, WB5/10; *n* = 8 per groups) until sacrifice for vascular casting with a zirconium dioxide-based contrast agent. Taking advantage of its absorption discontinuity at the K-absorption edge, vascular and bone images in the drill-hole defects were acquired by synchrotron radiation subtraction CT. Bone repair was reduced in HU rats. The bone volume fraction (B.Vf) was 88% smaller in HU5 and 42% smaller in HU10 than in WB5/10. The bone segment densities (B.Seg) were 97% smaller in HU5 and 141% larger in HU10 than in WB5/10, and bone thickness (B.Th) was 38% smaller in HU10 than in WB10. The vascular volume fraction (V.Vf) was 35% and the mean vessel diameter (V.D) was 13% smaller in HU10 than in WB10. When compared according to categorized vessel sizes, V.Vf in the diameter ranges 20–30, 30–40, and >40 *μ*m were smaller in HU10 than in WB10, and V.Seg in the diameter range >40 *μ*m was smaller in HU10 than in WB10. In contrast, there was no difference in B.Vf between DFOHU5/10 and WB5/10 and in V.Vf between DFOHU10 and WB10, though B.Seg remained 86% smaller in DFOHU5 and 94% larger in DFOHU10 than in WB5/10, and B.Th and V.D were 23% and 14% lower in DFOHU10 than in WB10. Vessel size-specific V.Vf in the diameter ranges 10–20 and 20–30 *μ*m was larger in DFOHU5 than in HU5. In conclusion, the enhanced angiogenic ingrowth mitigates the reduction in bone repair during mechanical unloading.

## Introduction

The adverse effects of mechanical unloading on tissue repair in general (Delp 2008; Farahani and DiPietro 2008), and on bone fracture healing specifically (Kaplansky et al. 1991; Kirchen et al. 1995; Midura et al. 2006), are well documented. Thus, prolonged bed rest or physical immobility following bone fracture, especially after hip fractures in the elderly (Marks et al. 2003), is undesirable. In contrast, early posttreatment mobilization should be encouraged to harness the biomechanical stimulus-dependent bone regenerative capacity (Szczesny et al. 2010; Boerckel et al. 2012). Early mobilization is beneficial not only for fracture healing, but also for regaining prefracture functionality and reducing the risk of severe secondary complications that can lead to discharge to a nursing home or even in-hospital death (Kamel et al. 2003; Hung et al. 2012; Gill et al. 2013). Therefore, it is crucial to accelerate the process of bone repair while the patient remains under unloaded conditions soon after fracture treatments to allow the fracture site to achieve the structural resistance needed for mobilization earlier.

Vascular disruption accompanying fracture leads to hematoma formation, which recruits inflammatory and progenitor cells and provides an environment in which these cells secrete cytokines and growth factors (Carano and Filvaroff 2003; Schindeler et al. 2008). Hypoxia is a major driving force behind the recruitment of progenitor cells (Ceradini and Gurtner 2005) and the production of angiogenic factors, such as vascular endothelial growth factors (VEGFs), by promoting the accumulation of hypoxia-inducible factors (HIFs) (Semenza 2001; Komatsu and Hadjiargyrou 2004). The hypoxia-activated HIF/VEGF pathway is a critical mediator of angiogenesis, and its involvement in the outcome of fracture healing is widely recognized (Street et al. 2002; Towler 2007; Wan et al. 2008; Schipani et al. 2009). Angiogenesis facilitates the supply of oxygen and nutrients, and thereby encourages multiple cellular processes during bone regeneration and the continuation of angiogenesis itself (Fraisl et al. 2009; Lu et al. 2013). Angiogenic vasculature also serves as a conduit for the invasion of osteoblast and osteoclast progenitors into the fracture site (Kindle et al. 2006; Towler 2008; Maes et al. 2010). Most recently, HIF-1*α*, one of the three HIF-*α* subunits, has been found to increase the proliferation of type H endothelial cells, which are a rich source of several growth factors relevant to the survival and proliferation of perivascular osteoprogenitors (Kusumbe et al. 2014).

Impaired angiogenesis is likely involved in the underlying mechanism resulting in the decreased osteogenic potential during skeletal unloading conditions (Sakuma-Zenke et al. 2005; Vandamme et al. 2012; Matsumoto et al. 2013). The reduced expressions of HIF-1*α* and several key angiogenic factors in unloaded skeletal muscles have also been reported (Wagatsuma 2008; McCabe et al. 2013). However, there has been no detailed quantification of angiogenic vascular structure during bone repair under skeletal unloading. Furthermore, the effects of stimulating angiogenesis on bone repair during skeletal unloading have not been fully explored. Thus, in this study, we quantified vascularization and bone formation in early-stage bone defect repairs in rats subjected to hindlimb unloading (HU), normal weight bearing (WB), or HU with administration of deferoxamine (DFO), a HIF/VEGF pathway activator (Koh and Powis 2012), and thereby tested the hypothesis that enhanced angiogenesis mitigates the unloading-induced reduction in bone repair. It has been reported that DFO leads to a positive outcome for both endochondral and intramembranous bone regeneration (Wan et al. 2008; Shen et al. 2009; Stewart et al. 2011; Farberg et al. 2012; Donneys et al. 2013; Grewal et al. 2013) although its effectiveness against unloading-induced reduction in bone healing has not been experimentally confirmed. Using a contrast agent containing zirconium dioxide particles (ZrCA) for vascular casting and taking advantage of its absorption discontinuity, three-dimensional images of angiogenic vessels and newly formed bone were acquired by synchrotron radiation-based subtraction microcomputed tomography (SRS*μ*CT), a method that was established in a previous study from our group (Matsumoto et al. 2013).

## Materials and Methods

Experiments were conducted according to the guiding principles of the American Physiological Society and with the approval of the Animal Research Committee of Osaka University Graduate School of Engineering Science. The detailed methods for SRS*μ*CT vascular and bone imaging and vascular casting with ZrCA were described previously (Matsumoto et al. 2013).

### Animals

Female Fischer rats aged 12 weeks (Clea Japan, Osaka, Japan) were anesthetized with an intraperitoneal dose of pentobarbital sodium (40 mg/kg). The skin over the medial aspect of the right lower leg was shaved, swabbed with povidone iodine, and incised. A full-thickness unicortical hole was created approximately 3 mm proximal to the tibio-fibula junction using a 0.7-mm diameter drill rotating at 183 s^−1^ (Muromachi Kikai, Kyoto, Japan). The drill margins were frequently irrigated with saline to avoid thermal necrosis, and the drill hole was flushed with saline to remove small bone fragments. After arresting bleeding from the bone marrow by applying pressure with a cotton swab, the skin was sutured and swabbed again. Each rat was housed alone with free access to standard food and water ad libitum in standard conditions of temperature (25 ± 1°C), humidity (60%), and light (12-h light-dark cycle). Buprenorphine (0.1 mg/kg) was administered subcutaneously as needed for pain relief.

The rats were divided into six groups: HU for 5 days (HU5, *n* = 8) or 10 days (HU10, *n* = 8), HU with DFO administration (DFOHU) for 5 days (DFOHU5, *n* = 8) or 10 days (DFOHU10, *n* = 8), and WB for 5 days (WB5, *n* = 8) or 10 days (WB10, *n* = 8). A tail-suspension procedure following the recommendations of Morey-Holton and Globus (2002) was implemented for the HU and DFOHU groups, with care taken to maintain a head-down tilt angle below 30°. A local injection of 20-*μ*L of DFO (200 *μ*mol/L; Sigma-Aldrich, St. Louis, MO) was given at the defect periphery from postoperative day 1 on alternate days (Shen et al. 2009) in the DFOHU5 (two doses) and DFOHU10 (five doses) groups. The other groups were similarly dosed on alternate days, but the DFO was replaced by the same volume of saline.

To determine the involvement of stress induction or increased sympathetic neural output involving *β*-adrenergic signaling in the HU-induced reduction in bone healing, an additional group of HU rats was similarly prepared and treated with *β*-adrenergic blockade for 10 days (PROHU10, *n* = 8). Propranolol (Sigma-Aldrich, St. Louis, MO) was administered as a nonselective blocker of *β*-adrenoreceptors via drinking water at a concentration of 0.5 g/L, which was reported to be effective at preventing bone loss in a tail-suspended rat model (Levasseur et al. 2003).

### Vascular casting

The rats were anesthetized with pentobarbital sodium again on postoperative day 5 (HU5, DFOHU5, WB5) or 10 (HU10, DFOHU10, WB10, PROHU10). The abdominal wall was incised, and the femoral artery and vein on the left side (opposite to the side of the hindlimb drill-hole surgery) were exposed by blunt dissection and ligated. The body was kept warm using an infrared heater (HIR-227; Omron, Kyoto, Japan), and the gut was moistened with warmed saline as needed. After heparin injection (1000 U) via the left femoral vein, the abdominal aorta and vein were exposed and ligated distal to the renal branches. The aorta was then cannulated in an antegrade manner distal to the ligation with a polyethylene catheter, and the vein was incised proximal to the femoral junction to allow drainage of blood and the infused solutions.

After euthanasia by pentobarbital sodium overdose, the right hindlimb was first flushed with 50 mL of heparinized saline (37°C, 100 U/mL) and then with 5 mL of 0.1 mol/L phosphate buffer (37°C) to avoid any adverse effects of the saline on ZrCA. Next, the hindlimb vasculature was gravity perfused at 120 mmHg with ZrCA (40°C), a 1.2-*μ*m-filtered mixture of 30 mL of 1.2% w/v agarose (Super LM; Nacalai Tesque, Kyoto, Japan) in 0.1 mol/L phosphate buffer and 15 mL of colloidal zirconium dioxide (ZR40BL; Nissan Chemical Industries, Tokyo, Japan). The latter was a water-based colloidal suspension containing 0.07 to 0.1 *μ*m particles of zirconium dioxide at a concentration of 62% w/v. The end point of the perfusion was determined by the appearance of superficial vessels in the femoral musculature and the purity of the outflow. Once perfusion was completed, the femoral artery and vein on the right side were ligated, and the entire body was immersed in an ice-cold water bath for approximately 1 h and stored overnight at 4°C. Each right tibia was then harvested and soaked in 4% paraformaldehyde at 4°C for 7 days. The diaphyseal segments with the drill-holes were removed with a low-speed diamond wheel saw (SBT650; South Bay Technology, San Clemente, CA) and sealed in polyethylene tubes containing 4% paraformaldehyde.

### SRSμCT

**Figure 1:**
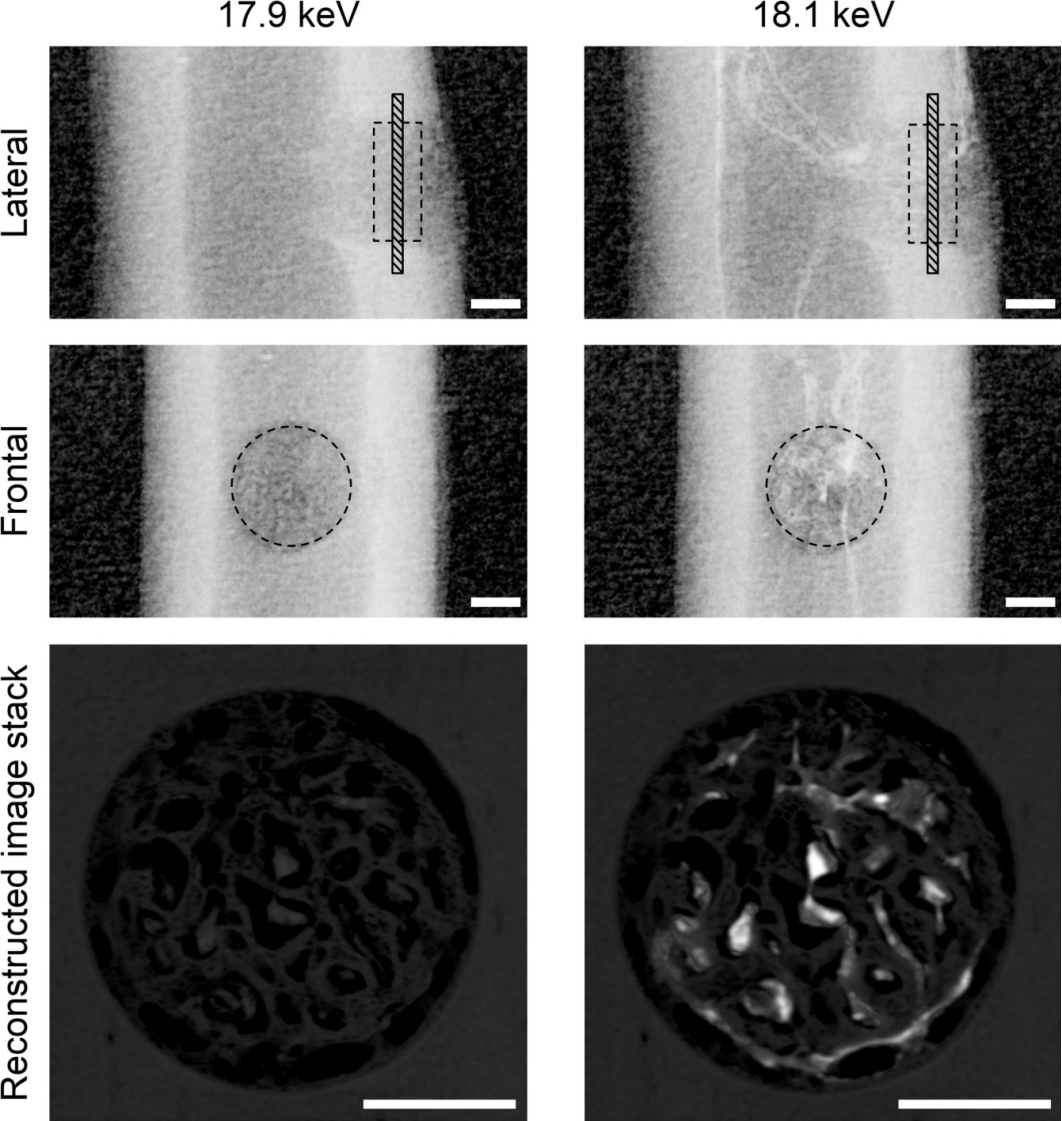
Lateral and frontal radiographs and stacks of reconstructed images of the drill-hole defect from a WB10 rat, obtained from synchrotron radiation at 17.9 keV (left) and 18.1 keV (right). The reconstructed image stacks comprise twenty 2.74-*μ*m-thick slices perpendicular to the drill-hole direction (hatched regions in the lateral radiographs). The enhanced contrast between blood vessels and bone on the 18.1-keV images is caused by the sharp absorption jump of ZrCA at just above its K-edge. The intercortical dashed circles and squares indicate the drill-hole defect region for analysis. Bar: 300 *μ*m.

At beamline 20B2 in the synchrotron radiation facility SPring-8 (Harima, Japan), the specimens were scanned by high-resolution CT using monochromatic synchrotron radiation just above (18.1 keV) and below (17.9 keV) the K-absorption edge of ZrCA. The synchrotron radiation was transformed into the visible spectrum through a 10-*μ*m-thick phosphor screen (Gd_2_O_2_S:Tb^+^) and detected using a cooled CCD camera equipped with a beam monitor (C4880-41S:AA40P; Hamamatsu Photonics, Hamamatsu, Japan). The sharp absorption jump in ZrCA at its K-absorption edge highlights vascular structures during 18.1-keV scanning (Fig.1), allowing selective visualization of the vasculature after image subtraction. Images of each specimen over an angular range 0–180° at 0.2° steps, and the light beam alone at 1° steps for calibration to compensate for light source instability were acquired at 1 sec exposure per frame. For background correction, 10 images of CCD dark current were also acquired at the start and end of each scan. Each scan dataset was reconstructed with a two-dimensional filtered backward projection algorithm using custom-built software, providing contiguous two-dimensional images composed of 2.74-*μ*m cubic voxels with 8-bit gray-scale resolution (Fig.1).

Prior to image subtraction for vascular region extraction, the gray values were first adjusted such that the voxel values of the linear absorption coefficient, *μ* (/cm), were given by 0.392 × gray value (0[black]–255[white]), resulting in gray values ranging from 0 to 255 for the 18.1-keV images and from 0 to approximately 60 for the 17.9-keV images. Next, pairs of 18.1- and 17.9-keV image stacks were corrected for misalignment arising mainly from the return-to-origin error of the CT stage using mutual information-based image registration (Maes et al. 1997). Then, to reduce the partial-volume effect, which can leave unwanted residuals near bone boundaries in the subtracted images even after fine alignment, bone regions in the 17.9-keV image stacks were expanded by three-dimensional maximum filtering.

Following these image preprocessing steps, the 17.9-keV image stacks were then subtracted from the 18.1-keV image stacks, and the subtracted image stacks were filtered with 3 × 3×3 voxel averaging and the vascular region was segmented from the rest using minimum cross-entropy thresholding (Li and Lee 1993). Finally, the original 17.9-keV image stacks were filtered by 3 × 3×3 voxel averaging, and all regions ≥4.74 per cm, except the vascular regions, were classified as regenerated bone. This threshold value of *μ* for bone segmentation corresponds to the density of hydroxyapatite, d.HAp, of 500 mg/cm^3^, determined by the relation: d.HAp (mg/cm^3^) = 136 × *μ* (/cm)* *− 142 = 7.97 ×  gray value − 142 (*r*^2^* *>* *0.999), which was derived from the 17.9-keV scan data of hydroxyapatite phantoms made from K_2_HPO_4_ solutions of various concentration. 11,000 1.46k software with a custom C program was used for image processing.

### Quantitative parameters for vascular and bone structures

**Figure 2:**
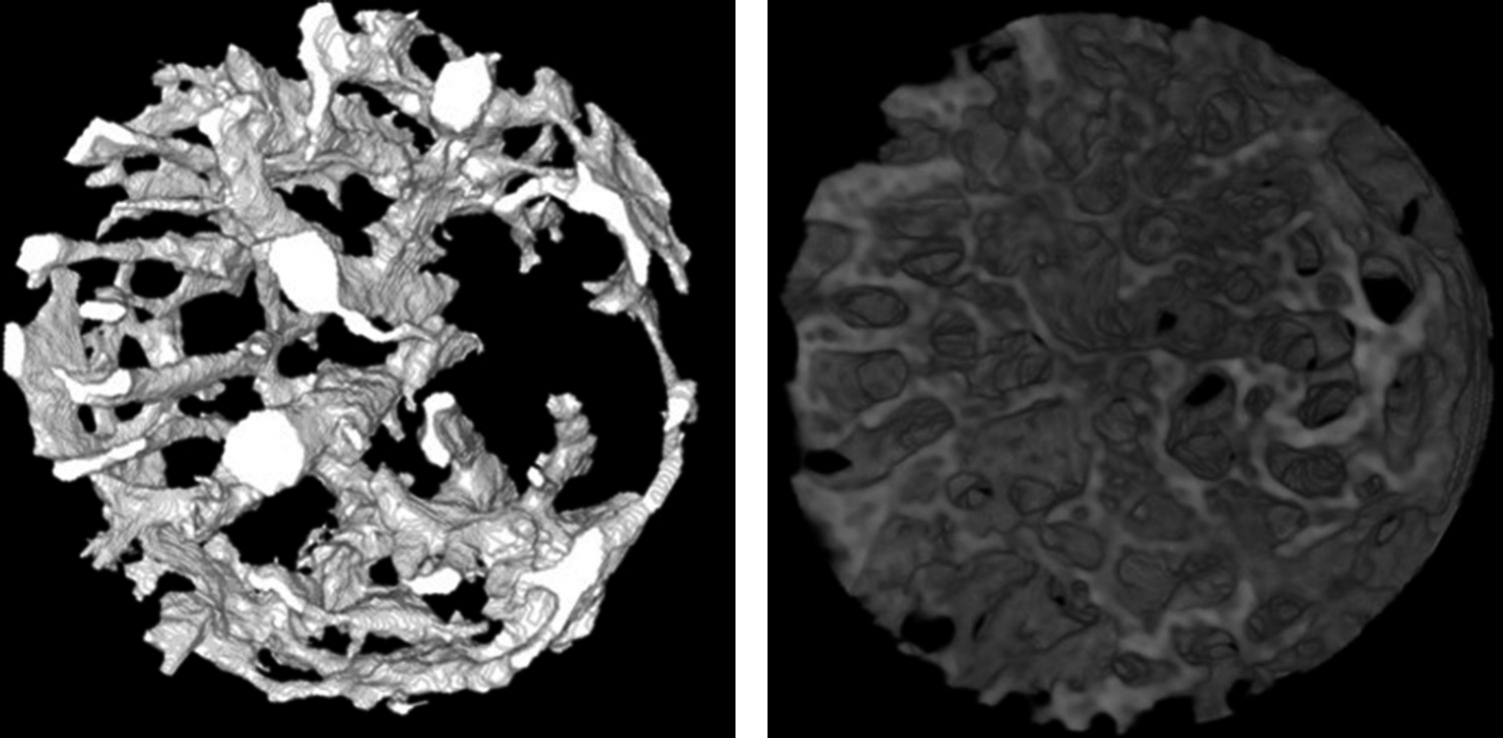
Three-dimensional displays of blood vessels (left) and bone (right) in a cylindrical region for analysis (diameter 720 *μ*m, height 300 *μ*m) from the cortical defect in Fig.1, produced by SRS*μ*CT. Bones were segmented as extravascular regions with the voxel intensity corresponding to d.HAp > 500 mg/cm^3^.

For structural analysis, a cylindrical region of interest was chosen within the cortical defect (Figs1 and 2). The vascular volume fraction, V.Vf (%); vessel diameter, V.D (*μ*m); the density of node-to-node or node-to-free end vessel segments, V.Seg (/mm^3^); bone volume fraction, B.Vf (%); bone thickness, B.Th (*μ*m); and the density of node-to-node or node-to-free end bone segments, B.Seg (/mm^3^); were calculated. The BoneJ plugin 1.3.5 for ImageJ 1.46k (Doube et al. 2010) was used to determine all indices except for V.D. Here, the local vessel diameter was defined as the diameter of the largest sphere falling inside the vascular space with its center on a vascular skeleton line given by a thinning algorithm, and V.D was defined as an average of these diameters over all skeleton lines. The diameter of each vessel segment was also determined as an average of local vessel diameters over its skeleton line, and vessel size-specific V.Vf and V.Seg were calculated for five divisions of vessels classified according to their diameters. Using the *μ*-d.HAp relation, local d.HAp values were determined for both newly formed bone within the defect and intact cortical bone at a diaphyseal site proximal to the defect in specimens in the HU10, DFOHU10, WB10, and PROHU10 groups.

### Statistics

Data are presented as mean ± standard error of the mean (SEM). Differences between HU5, DFOHU5, and WB5 or HU10, DFOHU10, WB10, and PROHU10 were assessed by Kruskal–Wallis tests followed by Dunn's multiple comparison tests because the data on structural indices frequently violated the assumptions of equal variances between groups or a Gaussian distribution. Body weight changes within the groups were assessed by paired *t*-test. We used Prism 6 (version 6.0b; GraphPad Software, San Diego, CA) for the statistical analysis. Values of *P *<* *0.05 were considered statistically significant.

## Results

Over the 5- and 10-day experimental periods, body weight decreased in the HU groups (*P *<* *0.01): from 141 ± 2 to 131 ± 3 g in HU5, from 144 ± 2 to 132 ± 1 g in DFOHU5, from 142 ± 2 to 133 ± 1 g in HU10, and from 146 ± 3 to 134 ± 1 g in DFOHU10. Body weight was unchanged (149 ± 2 vs. 148 ± 2 g) in WB5 and increased from 143 ± 2 to 148 ± 2 g (*P *<* *0.01) in WB10. On the day of sacrifice, the mean body weight was lower in groups HU5 (*P *<* *0.01) and DFOHU5 (*P *<* *0.01) than in WB5, and lower in groups HU10 (*P *<* *0.01) and DFOHU10 (*P *<* *0.05) than in WB10.

**Figure 3:**
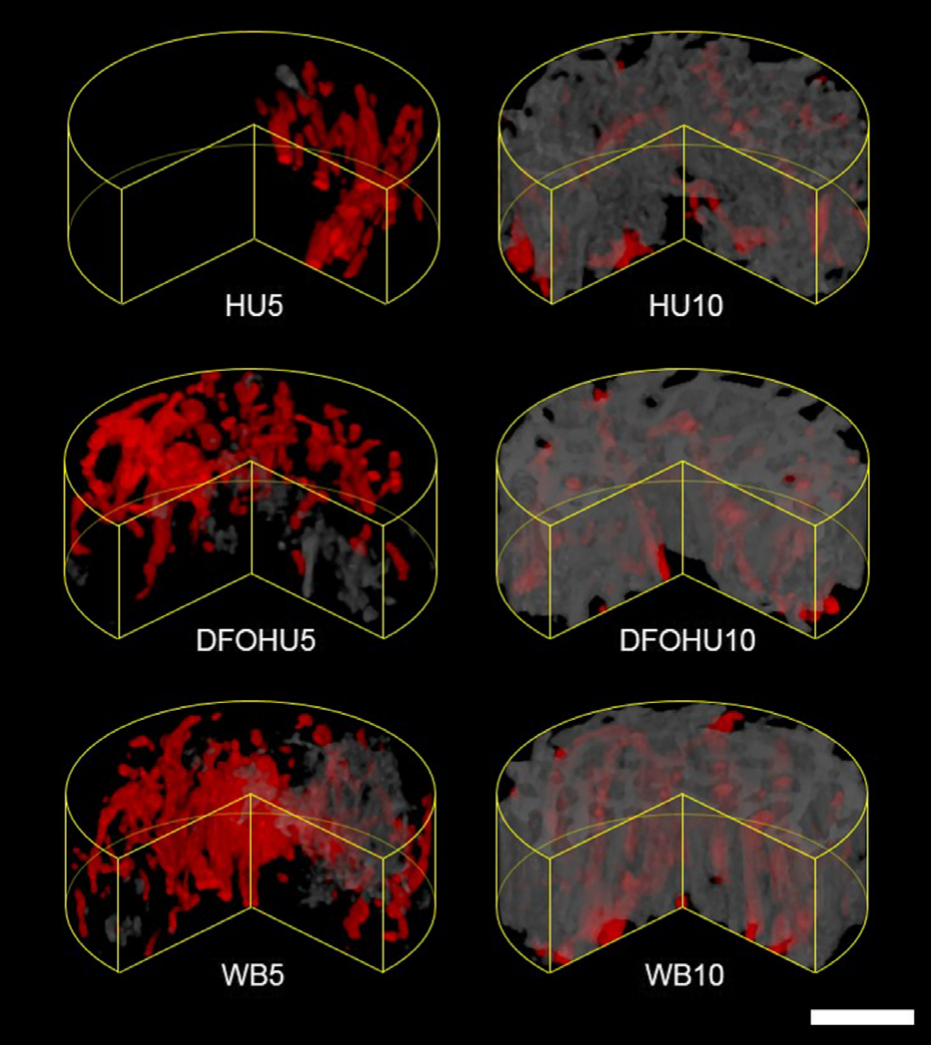
Volume-renderings of blood vessels (red) and bone (gray) within a defect region for each group. The anterior quarter sector was cut from each cylindrical region of interest to make the inner region more visible. Both bone formation and vascularization were considerably suppressed in HU5. Woven-like bone structures were observed in HU10, but comprising a larger number of small-sized bone segments compared with those in DFOHU10 or WB10. Bar: 200 *μ*m.

Typical images within the bone defects in each group are shown in Figure3. On postoperative day 5, angiogenesis and bone generation were observed, along with greater volumes of vasculature in every group. Compared with WB5 and DFOHU5, HU5 showed poor angiogenesis and markedly-reduced osteogenesis. By postoperative day 10, more bone generation had occurred than angiogenesis in every group, resulting in larger spaces occupied by new bone. Woven-like bone with a solid appearance was observed in both WB10 and DFOHU10. New bone structures in HU10 were also woven-like, but were composed of a large number of small, thin segments compared with those in DFOHU10 and WB10.

The bone and vascular structural indices are summarized in Table1. On postoperative day 5, HU-induced decrease in bone repair was found, and B.Vf and B.Seg were significantly smaller in HU5 than in WB5. These reductions were mitigated with an increasing trend in V.Vf by DFO administration (*P *=* *0.054, DFOHU5 vs. HU5), though B.Seg was still significantly smaller and B.Vf tended to be lower (*P *=* *0.11) than those in WB5. On postoperative day 10, HU treatment influenced most structural indices, resulting in significantly smaller B.Vf, B.Th, V.Vf, and V.D and larger B.Seg in HU10 than those in WB10. The HU-induced decrease in bone repair was mitigated by DFO administration, which also improved angiogenesis. No differences were found in B.Vf and V.Vf between DFOHU10 and WB10, though V.D and B.Th remained smaller and B.Seg was significantly larger in DFOHU10.

**Table 1:** Bone and vascular structural indices

	Postoperative day 5			Postoperative day 10		
	HU5	DFOHU5	WB5	HU10	DFOHU10	WB10
B.Vf (%)	0.18 ± 0.08**	0.54 ± 0.18	1.44 ± 0.33	26.15 ± 2.40**	37.14 ± 2.43	45.32 ± 2.11
V.Vf (%)	4.48 ± 1.66	9.08 ± 1.17	6.57 ± 1.60	4.61 ± 0.63*	6.05 ± 0.32	7.06 ± 0.77
B.Th (*μ*m)	15.08 ± 2.67	16.84 ± 2.79	11.80 ± 0.59	21.59 ± 1.30**	26.89 ± 1.63*	34.81 ± 1.18
V.D (*μ*m)	20.20 ± 2.22	20.93 ± 1.04	19.64 ± 0.87	17.25 ± 0.77*	18.07 ± 0.32*	19.87 ± 0.35
B.Seg (/mm^3^)	117 ± 57**	470 ± 223*	3388 ± 986	40413 ± 3437**	32531 ± 3197*	16777 ± 808
V.Seg (/mm^3^)	1796 ± 621	2911 ± 387	2401 ± 615	2183 ± 231	2830 ± 255	2873 ± 458

Values are presented as mean ± SEM.

*P <* *0.05, **P <* *0.01 versus WB5 or WB10.

Tables2 and 3 shows vessel size-specific V.Vf and V.Seg for five vessel diameter ranges. On postoperative day 5, V.Vf in HU5 and DFOHU5 did not differ from that in WB5 over all diameter ranges, but V.Vf was larger in DFOHU5 than in HU5 in the diameter ranges 10–20 and 20–30 *μ*m. On the other hand, no difference was found in V.Seg between DFOHU5 and WB5 over all diameter ranges. On postoperative day 10, V.Vf in the diameter ranges 20–30, 30–40, and >40 *μ*m and V.Seg in the diameter range >40 *μ*m were smaller in HU10 than in WB10. Neither V.Vf nor V.Seg differed between DFOHU10 and WB10 over all diameter ranges.

**Table 2:** Vascular volume fractions (%) of different vessel diameters

Vessel diameter (*μ*m)	Postoperative day 5			Postoperative day 10		
	HU5	DFOHU5	WB5	HU10	DFOHU10	WB10
<10	0.03 ± 0.01	0.06 ± 0.01	0.04 ± 0.01	0.06 ± 0.02	0.04 ± 0.01	0.03 ± 0.01
10–20	1.23 ± 0.34	3.33 ± 0.55‡‡	2.42 ± 0.44	2.21 ± 0.16	2.58 ± 0.30	2.54 ± 0.38
20–30	1.05 ± 0.37	2.93 ± 0.49†	2.05 ± 0.58	1.63 ± 0.30*	1.91 ± 0.20	2.85 ± 0.41
30–40	0.88 ± 0.35	1.59 ± 0.17	1.39 ± 0.40	0.44 ± 0.12**	0.76 ± 0.10	1.23 ± 0.14
>40	1.28 ± 0.99	1.16 ± 0.27	0.67 ± 0.25	0.04 ± 0.03**	0.11 ± 0.03	0.41 ± 0.14

Values are presented as mean ± SEM.

*P <* *0.05, **P <* *0.01 versus WB10; ^†^*P *<* *0.05, ^††^*P *<* *0.01 versus HU5.

**Table 3:** Density of vessel segments (/mm^3^) of different vessel diameters

Vessel diameter (*μ*m)	Postoperative day 5			Postoperative day 10		
	HU5	DFOHU5	WB5	HU10	DFOHU10	WB10
<10	73.5 ± 18.6	119.7 ± 22.5	84.9 ± 23.7	114.9 ± 31.6	73.8 ± 17.1	68.5 ± 17.9
10–20	838.6 ± 301.8	1516.3 ± 239.0	1225.3 ± 258.4	1290.4 ± 95.0	1715.9 ± 179.9	1453.3 ± 220.3
20–30	453.6 ± 182.0	747.7 ± 126.6	602.5 ± 195.5	581.9 ± 97.2	728.3 ± 63.5	898.7 ± 157.8
30–40	225.7 ± 90.5	329.6 ± 31.3	329.6 ± 110.8	182.4 ± 54.2	265.6 ± 31.6	363.7 ± 56.4
>40	204.9 ± 137.2	197.4 ± 36.0	159.1 ± 69.9	13.7 ± 12.5*	46.4 ± 14.6	89.2 ± 36.1

Values are presented as mean ± SEM.

* *P *<* *0.05 versus WB5 or WB10.

**Figure 4:**
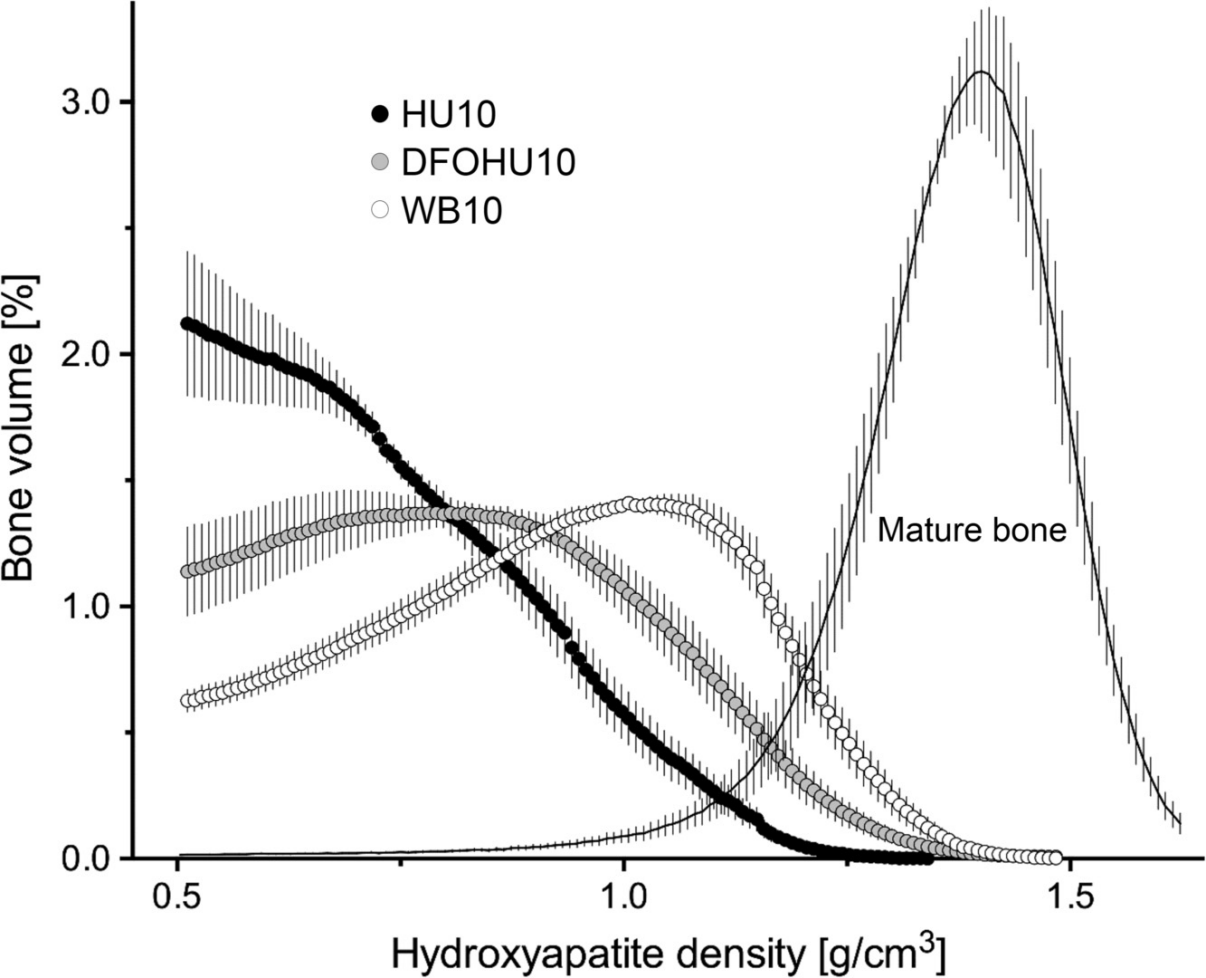
Relative distribution of bone volume versus density of hydroxyapatite (d.HAp) within the defect region from HU10, DFOHU10, and WB10. The d.HAp distribution in the intact diaphyseal cortical segment over all three groups is also shown. The bone volume at each d.HAp value (bin width: 8 mg/cm^3^) is expressed as a percentage of the total volume of voxels where d.HAp is >500 mg/cm^3^. Values are mean ± SEM.

The relative distributions of bone volume versus d.HAp within the defects of HU10, DFOHU10, and WB10 and in the intact cortical region of the tibial diaphyses all in the three groups are shown in Figure4. At this stage, the mineralization of the newly formed bone in the defect was immature in every group. The mean (median) values of d.HAp were 737 ± 16 (718 ± 18) in HU10, 834 ± 19 (830 ± 23) in DFOHU10, and 919 ± 15 (938 ± 19) in WB10, and 1364 ± 15 (1377 ± 15) mg/cm^3^ in intact cortical bone. Both the mean and median values differed significantly between HU10 and WB10 (*P *<* *0.05), while neither of them in DFOHU10 differed from those in WB10, showing the moderating effect of DFO on the HU-induced delay of mineralization.

**Figure 5:**
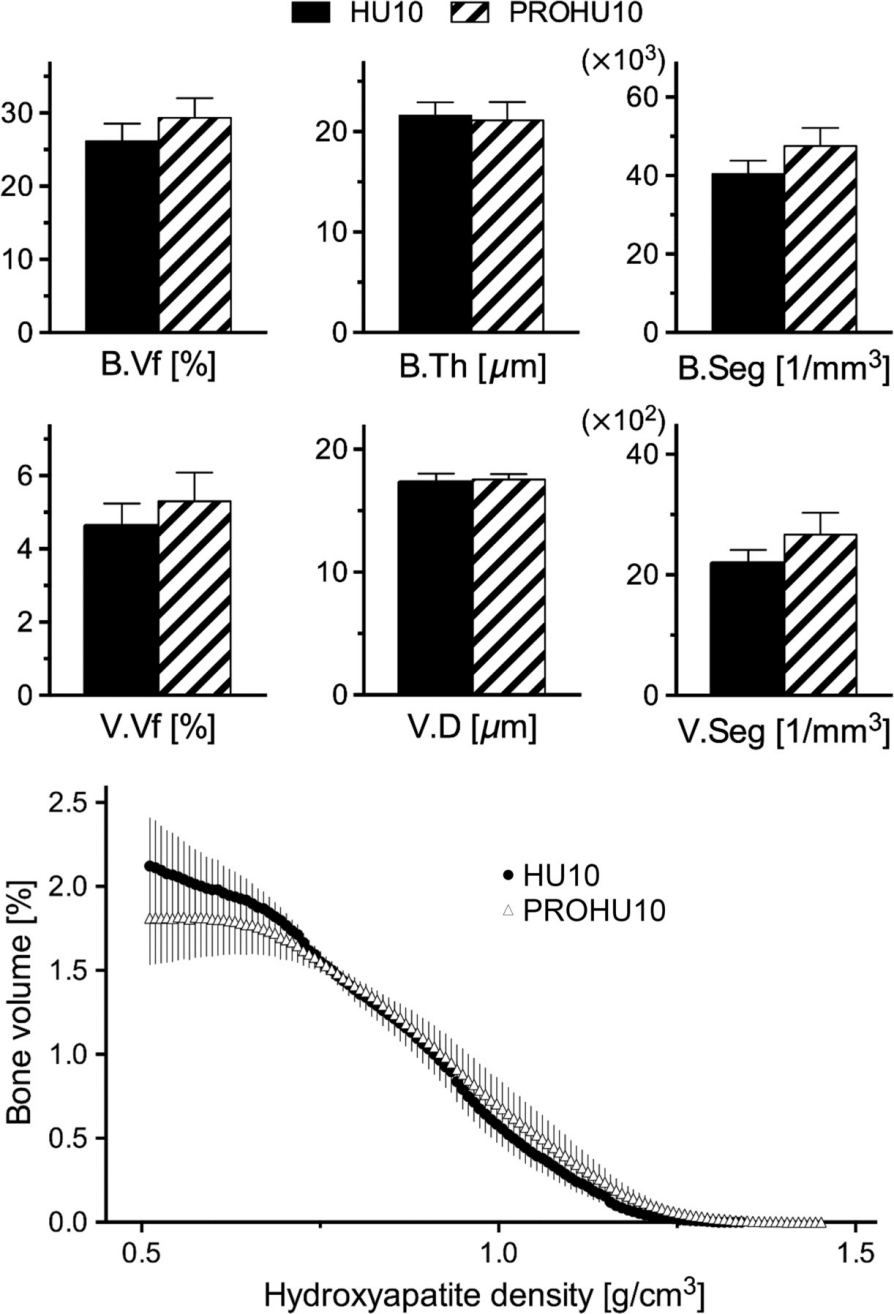
No differences were found between HU10 and PROHU10 in bone and vascular volume fractions (A), bone thickness and vascular diameter (B), bone and vessel segment densities (C), or the relative distribution of bone volume versus d.HAp (D), shown in the same manner as in Fig.4. Bars represent mean ± SEM.

The 10-day treatment with *β*-adrenergic blockade did not alter the HU effect on bone repair and body weight gain. The bone and vascular structural indices and d.HAp distribution were similar between PROHU10 and HU10 (Fig.5). The body weight in PROHU10 decreased from 143 ± 1 to 130 ± 2 g over 10 days (*P *<* *0.01), and these values were not different from those in HU10.

## Discussion

The primary aim in this study was to determine whether enhanced angiogenesis can suppress the decrease in early-stage bone healing that is caused by simulated bedrest, physical inactivity, or microgravity. Our results based on SRS*μ*CT of rat tibial cortical defect repair are consistent with the hypothesis that the stimulation of angiogenesis mitigates the HU-induced reduction in bone repair capacity, suggesting the therapeutic potential of pro-angiogenic agents for bone healing during skeletal unloading.

We used a drill-hole model of defect repair in the tibial diaphyseal bone of rats, which provides a defect site with mechanical stability and leads to bone arising de novo without the need for a preexisting cartilage framework (intramembranous ossification) (Thompson et al. 2002; Monfoulet et al. 2010; He et al. 2011). Using the combined techniques of SRS*μ*CT and ZrCA for vascular casting, we identified the HU-induced suppression of bone regeneration and the inhibitory effect of HU on angiogenesis during the early stages of bone repair. Previous studies have shown the histological appearance of reduced angiogenesis during cutaneous wound healing under HU (Radek et al. 2008) and during osteotomy healing under authentic microgravity (Kirchen et al. 1995). In the latter study, however, no evidence for an antiangiogenic effect of HU was found. To the best of our knowledge, this study is the first to demonstrate the HU-induced suppression of angiogenesis during bone repair.

Angiogenesis during bone healing is primarily governed by the typically low oxygenation state of the tissue, but its progression is also influenced by HU. The reduction in mechanical stimuli caused by removal of the ground reaction force and hypokinesia/hypodynamia of the hindlimbs attenuates the angiogenic capacity of the fracture hematoma by decreasing VEGF levels (Groothuis et al. 2010). The loss of body weight associated with HU suggests a decrease in serum leptin (Baek et al. 2008), a proangiogenic factor derived mainly from peripheral adipose tissue (Cao et al. 2001). Lymphatic dysfunction during HU (Gashev et al. 2006) could potentially reduce the reservoir of angiogenic growth factors by limiting the migration of lymphocytes and plasma cells to the defect (Radek et al. 2008). Indeed, HU has been reported to decrease the expression of angiogenic factors (Wagatsuma 2008; McCabe et al. 2013). Furthermore, the reduction in blood flow to the unloaded hindlimbs (Colleran et al. 2000) will diminish endothelial cell survival (Melchionna et al. 2005). These antiangiogenic effects result in decreases in endothelial-derived bone growth factors such as bone morphogenetic proteins (BMPs) (Kasperk et al. 1997; Mundy et al. 2001; Lin and Hankenson 2011). Consequently, osteoblastic differentiation and proliferation is likely downregulated, and the level of osteoblast-derived VEGF for paracrine stimulation of endothelial cell proliferation, migration, and tube formation (Deckers et al. 2002; Clarkin et al. 2008) will also be reduced. Such HU-associated alterations, together with the angiogenic stimulation via gait activity that was started soon after the defect creation in the WB group (Moore et al. 2003; Boerckel et al. 2011), could account for the reduced angiogenesis during bone repair as shown by lower V.Vf and V.D in HU10 than in WB10 (Table1). The significant differences in vessel size-specific V.Vf and V.Seg between HU10 and WB10 observed in the large diameter ranges (Tables2 and 3) imply the inhibitory effect of HU against the ingrowth of angiogenic vessels.

The adverse effects of HU on bone repair are not derived exclusively from poor angiogenesis. However, angiogenesis is indispensable for bone regeneration (Hausman et al. 2001; Street et al. 2002) and vascular ingrowth into the fracture site encourages osteogenesis by sustaining a high metabolic activity in osteoblasts engaged in bone repair, while also serving as a migration route for osteoprogenitors into the injured zone (Towler 2008; Maes et al. 2010). Thus, it is very plausible that the observed decrease in angiogenesis is involved in the HU-induced reduction in bone repair, and that angiogenic stimulation is effective at mitigating the reduction in bone repair. One of the most effective substances for stimulating angiogenesis is DFO because it could enhance angiogenesis to the same level observed in genetic models with HIF-1*α*-overexpressing osteoblasts (Wan et al. 2008). It has been reported that DFO leads to a positive outcome for healing of weight-bearing bone (Wan et al. 2008; Shen et al. 2009; Stewart et al. 2011; Grewal et al. 2013) although its effectiveness against unloading-induced reduction in bone healing has not been confirmed. Furthermore, DFO remains effective in improving detriments of angiogenesis and osteogenesis in non–weight bearing bones such as mandible (Farberg et al. 2012; Donneys et al. 2013). Thus, we anticipate that DFO mitigates the reduction in defect repair associated with compromised angiogenesis in weight-bearing bone placed under unloaded conditions. Actually, DFO administration to the HU rats increased the vessel size-specific V.Vf (10–30 *μ*m) on postoperative day 5 and reduced the HU-mediated decreases in V.Vf and vessel size-specific V.Vf (>20 *μ*m) and V.Seg (>40 *μ*m) on postoperative day 10. These DFO effect on angiogenic vascularity would contribute to mitigating the adverse effect of HU on bone defect repair.

However, the 10-day DFO treatment did not restore the HU-induced reduction in vascularization to the level observed in WB10, as shown by smaller V.D and, although not statistically significant, trends toward smaller vessel size-specific V.Vf and V.Seg in large diameter ranges in DFOHU10 compared with those in WB10. This incomplete restoration may be associated with the smaller B.Th and larger B.Seg in DFOHU10, creating a delicate, woven-like bone structure. In this study, the administration regimen of DFO was not optimized, and modifying the dosing regimen could further enhance the proangiogenic effects of DFO. Nonetheless, the use of DFO alone may not be able to overcome the reduction in bone repair during HU because other factors not involved with angiogenesis may contribute to the HU-induced reduction in bone repair. Decreased osteoblastic differentiation and proliferation attributed directly to the reduction in mechanical stimuli (Keila et al. 1994) may substantially inhibit bone repair. In addition, reductions in arterial pressure and blood supply to the hindlimbs (Colleran et al. 2000; Stabley et al. 2013) might decrease the recruitment of neutrophils and inflammatory macrophages from the vascular space to the defect site during the inflammatory phase (Radek et al. 2008; Schindeler et al. 2008; Claes et al. 2012), possibly delaying the process of bone repair.

Stress induction or increased *β*-adrenergic signaling could be inferred from the lack of weight gain for the rats with HU treatment. This could be a potential antiosteogenic factor during HU other than reduced angiogenesis because *β*-adrenergic stimulation reduces the osteogenic capacity by suppressing osteoblastic activity and osteoblastic differentiation directly or indirectly via peripheral leptin reduction (Levasseur et al. 2003; Kondo et al. 2005; Hamrick and Ferrari 2008; Marenzana and Chenu 2008; Baek and Bloomfield 2009). However, the nonselective *β*-blockade did not improve the HU-induced impairment of bone repair as well as angiogenesis (Fig.5). Thus, the reduction in bone repair observed in the present HU rat model is unlikely to involve osteo-regulatory actions of *β*-adrenergic signaling. The functions of osteoblasts may also be modulated by *α*-adrenergic signaling, though its expression in bone cells remains controversial (Elefteriou et al. 2005). However, *α*-adrenergic stimulation would be expected to improve rather than further decrease the reduced bone healing during HU because of its stimulation of osteoblastic proliferation (Huang et al. 2009), leading to an increase in osteogenic capacity.

Barium sulfate-based contrast agents have been predominantly used for *μ*CT imaging of bone vasculature without decalcification because barium sulfate is available in high concentration and provides good contrast between the vasculature and bone, especially when using synchrotron radiation (Fei et al. 2010; Roche et al. 2012). However, angiogenic vessels in the defect, unlike mature vessels, have irregular contours and do not exhibit tree-like interconnections (Fig.2), likely leading to uneven vascular filling with contrast materials and possibly yielding a vascular cast with local radiopacity comparable to bone. Although increasing the contrast-material concentration raises the level of vascular radiopacity and permits image segmentation into angiogenic vasculature and regenerated bone by setting a higher threshold value, the inevitable increase in viscosity reduces microvascular perfusability and can increase the incomplete vascular filling. In contrast, the viscosity of the novel contrast agent (ZrCA) is similar to the apparent blood viscosity in microvasculature (Matsumoto et al. 2013). Thus, using ZrCA for vascular casting and taking advantage of its K-absorption edge, we were able to determine the angiogenic vascular structure as radiointensity-enhanced regions while minimizing uneven vascular filling artefacts. In addition, the energies close to the K-absorption edge of ZrCA are well suited for quantification of bone mineral density in small or rodent bone specimens.

Besides not determining the most effective DFO administration protocol, there are several limitations to this study. First, as a potent iron chelator, DFO possibly contributes to the enhancement of bone regeneration through the direct stimulation of osteoblastic differentiation (Qu et al. 2008; Chen et al. 2014; Chung et al. 2014). The degree of contribution of the angiogenic vs. direct osteogenic actions of DFO on bone regeneration remains to be determined in future studies. The efficacy of DFO in bone defect repair during weight bearing will be also worth investigating. Second, in most clinical cases, bone repair occurs through a mixed process of intramembranous and endochondral bone formation. In the latter, the bone regeneration process arises from a preexisting cartilage framework in a mechanically unstable state. Thus, the effects of DFO should be confirmed in bone repair using a more clinically-relevant animal model. Third, the CT scan datasets were acquired from postmortem specimens, and the evaluations were made at only two time points during early-stage bone repair. Further evaluation requires more scans at different time points over the early-to-late stages of bone repair especially for the analysis of vascular ingrowth and remodeling in fracture. Considering the number of animals sacrificed, low-dose CT, which allows longitudinal scanning of the same living animal, should be suitable for longer-term analyses, though it does entail the administration of nontoxic contrast agents appropriate for bone microvascular imaging.

## Conclusions

In conclusion, using the combined techniques of SRS*μ*CT and ZrCA for vascular casting, we have demonstrated the potential of stimulating angiogenesis with DFO to mitigate the HU-induced reduction in early-stage defect repair in rat cortical bone. The present DFO dosage, however, did not completely restore bone repair capacity of HU rats to the levels observed in WB rats. Direct osteoblastic stimulation with factors that replace mechanical stress together with stimulation of angiogenesis using DFO administration regimens that are more effective may further increase bone repair during skeletal unloading.
